# Acute toxicity and the 28-day repeated dose study of a Siddha medicine Nuna Kadugu in rats

**DOI:** 10.1186/1472-6882-12-190

**Published:** 2012-10-22

**Authors:** Ramaswamy Selvaratnam Ramaswamy, Nettam Prathyusha, Ruthiramoorthi Saranya, Haridass Sumathy, Kutuva Tulasi Mohanavalli, Raju Jyothi Priya, Jayakothanda Ramaswamy Venkhatesh, Chidambaram Saravana Babu, Kumarasamy Manickavasakam, Sadagopan Thanikachalam

**Affiliations:** 1 Department of Sirappu Maruthuvam, National Institute of Siddha, Tambaram Sanatorium, Chennai, 600 047, India; 2 Centre for Toxicology and Developmental Research (CEFT), Sri Ramachandra University, Ramachandra Nagar, Chennai, 600 116, India

**Keywords:** Morinda Pubescens, Nuna, Nuna Kadugu, Vitiligo, Acute toxicity, Sub-acute toxicity

## Abstract

**Background:**

Nuna Kadugu (NK), a Siddha medicine prepared from leaves and fruits of *Morinda Pubescens*, used for the treatment of various skin diseases. Though NK has been widely used for several decades, no scientific report was available on its safety. Present study was undertaken to demonstrate the oral toxicity of NK in Sprague Dawley rats.

**Methods:**

Acute and 28-day repeated oral toxicity studies were performed following OECD test guidelines 423 and 407, respectively, with minor modifications. In acute oral toxicity study, NK was administered at 2000mg/kg b.wt., p.o and animals were observed for toxic signs at 0, 0.5, 1, 4, 24 h and for next 14 days. Gross pathology was performed at the end of the study. In repeated dose, the 28- day oral toxicity study, NK was administered at 300, 600 and 900 mg/kg b.wt./p.o/day. Two satellite groups (control and high dose) were also maintained to determine the delayed onset toxicity of NK. Animals were observed for mortality, morbidity, body weight changes, feed and water intake. Haematology, clinical biochemistry, electrolytes, gross pathology, relative organ weight and histopathological examination were performed.

**Results:**

In acute toxicity study, no treatment related death or toxic signs were observed with NK administration. In the repeated dose study, no significant differences in body weight changes, food / water intake, haematology, clinical biochemistry and electrolytes content were observed between control and NK groups. No gross pathological findings and difference in relative organ weights were observed between control and NK treated rats. Histopathological examination revealed no abnormalities with NK treatment.

**Conclusion:**

Acute study reveals that the LD_50_ of NK is greater than 2000mg/kg, b.wt. in fasted female rats and can be classified as Category 5. 28-day repeated oral toxicity demonstrates that the No Observed Adverse Effect Level of NK is greater than 900 mg/kg b.wt./day, p.o in rats. There were no delayed effects in NK satellite group. In conclusion, NK was found to be non-toxic in the tested doses and experimental conditions.

## Background

Skin diseases are one among the major diseases for which traditional medicines are preferred and largely utilized. Vitiligo or leucoderma is characterized by skin depigmentation resulting in the appearance of patches due to melanocyte dysfunction. The exact pathogenesis of vitiligo remains unclear and is likely to be multifactorial, suggesting the involvement of autoimmune and genetic factors, oxidative stress, neural, or viral sources [1]. Though the prevalence of vitiligo varies significantly with age, there is no significant difference seen between genders [2]. The incidence of vitiligo is 0.1-2% worldwide and 0.25 - 2.5% in India [2,3]. Vitiligo is termed as “Venpulli Noi” or “Venpadai” in Siddha system of medicine. On the basis of three vital humours, Siddha system classifies vitiligo (venpadai) as “Vatha venpadai”, “Pitha venpadai” and “Kaba venpadai”. Another type of vitilgo is called “Megha venpadai” (through sexual transmission) in the Siddha pathophysiology of vitiligo.

*Morinda Pubescens* (Family: Rubiaceae) commonly known as Indian Mulberry, is an evergreen shrub or small tree of 5–10 m tall and a promising medicinal plant used widely by the Siddha practitioners [4]. It is an indigenous plant of Tamilnadu and is called “Nuna” in Tamil. Ethnomedically, the bark of Nuna is well known for its use in the treatment of eczema, fever due to primary complexes, ulcers and glandular swellings; whilst the leaves are useful in digestive disorders (Mantham) in children and in venereal diseases [5]. Fruit extract of Nuna is reported to possess antimicrobial, antifungal, wound healing, antidiabetic and hepatoprotective activities [4,6,7].

Pattai karappanodu paarachileshma suram

Ottinindra pun kranthi ottunkaan – Mattalarai

Yenthu Nunaavin ilai mantham theerthu nalla

Kaanthi tharu meham adunkaan

- Agathiyar Gunavakadam

The meaning of this Siddha verse is that the bark of Nuna is useful in the treatment of eczema (karappan), fever (Iyya suram), ulcers, and tumours/swellings (kranthi). Leaves relieve indigestion (mantham), venereal diseases (Mega noikal) and also give luster to skin. Medicinal preparations using Nuna leaves and gingelly oil called as Nuna kadugu (internal use) and Nuna thailam (external application) are indicated for the treatment of vitiligo [8].

An open label clinical study (40 participants) with Nuna kadugu (NK) reveals its evidence-based use in the treatment of vitiligo (our unpublished data), however there is no scientific report to evaluate potential human toxicity. Hence, the present study was undertaken to establish the toxicity profile of NK in experimental animals which will render strong evidence for its safety in clinical use.

## Methods

### Chemicals and reagents

Clinical diagnostic kits were purchased from M/s. Accurex Biomedical Pvt. Ltd., India. All other chemicals and reagents used were of analytical grade.

### Plant material

Leaves and fruits of *Morinda pubescens* (Nuna) were procured from M/s. Arogya Health Care Pvt. Ltd., Chennai, traditional herbs and raw drugs dealer. Materials were authenticated by Dr. Sasikala Ethirajulu, Assistant Director (Pharmacognosy), Siddha Central Research Institute, Chennai, India. Voucher specimen of the herbarium (NIS/SM-Dept/herbarium/2010/Nuna/RAM) were prepared and stored in the Department of Sirappu Maruthuvam, National Institute of Siddha, Chennai, for future references.

### Preparation of medicine

The leaves and fruits of *Morinda Pubescens* (Nuna) were freed from earthy matters, decayed leaves, fruits and washed with water. They were chopped into small pieces and ground into a paste form called “Karkam” (as per the classical Siddha literature) by adding sufficient quantity of water in “Kalvam” (a traditional grinding tool made of stone). Karkam was weighed and boiled with equal weight of unrefined gingelly oil till it disintegrated to the size of mustard seeds called as “Nuna Kadugu”. It was filtered to remove the oil now called “Nuna Thailam” which was later cooled and stored. Nuna Kadugu was blotted, using butter paper, multiple times to remove the last traces of oil.

### Standardisation of nuna kadugu

#### Phytochemicals

Preliminary phytochemical qualitative screening of NK was performed using standard method [9]. Phytochemical constituents such as phenolic compounds, reducing sugars, flavones, glycosides, saponins, alkaloids, anthroquinones, proteins and tannins were qualitatively analyzed. Total phenols were tested by reacting NK with few drops of alcoholic ferric chloride solution. Flavones were tested by adding 10% sodium hydroxide solution or ammonia with NK. Glycosides were tested by mixing NK with a little anthrone on a watch glass and a drop of concentrated sulphuric acid and warming gently over water bath. Saponins were tested by shaking NK with water for frothing test. NK was tested for the presence of reducing sugars, alkaloids, anthraquinones, quinones, protein and tannins by reacting with Fehling’s solution A and B, acetic acid followed by Draggendroff’s reagent, aqueous ammonia or caustic soda, sodium hydroxide, few drops of biuret reagent and basic lead acetate solution respectively.

Content of total phenols in NK was determined by the method proposed in earlier method [10], Briefly, 1.25ml of 1:10 diluted Folin’s Ciocalteau reagent and 1ml of 7.5% Na_2_CO_3_ were added to tubes containing 0.25 ml of the NK (1mg/ml). The mixture was allowed to stand for 30 min at 37°C and the colour intensity was measured at 765 nm. Total phenol content was expressed in terms of %w/w. Tannins was performed as per the earlier method [11]. 0.2ml of the NK (0.5mg/ml) was made up to 0.5ml with methanol. 0.25 ml of Folin’s phenol reagent and 2.5 ml of 1% sodium carbonate were pipetted into all the tubes. The tubes were incubated for 5 minutes at room temperature. 0.25 ml of Folin’s phenol and 2.5 ml of 1% sodium carbonate serves as blank. The blue colour developed was measured at 640 nm. The content was expressed in %w/w. Finally flavonoid content of NK was employed using aluminium chloride colorimetric method [12]. To 1ml of NK (1mg/ml), 0.1 ml of 10% aluminum chloride, 0.1 ml of 1 M sodium acetate and 2.8 ml of distilled water were added. The tubes were incubated at room temperature for 30 min. The absorbance was measured at 415 nm. The content was expressed in %w/w.

#### Aflatoxins and heavy metals

Aflatoxins (B1, B2, G1 and G2) using LC-MS technique and heavy metals (lead, cadmium, mercury and arsenic) contents using atomic absorption spectroscopy were also determined following standard protocols.

#### HPTLC fingerprint of NK

High Performance Thin Layer Chromatography (HPTLC) fingerprint of NK was developed using Silica gel GF_254_ as stationary phase and toluene: ethyl acetate: formic acid: acetic acid (30:30:8:2.5) as mobile phase. The plates were developed in twin trough chamber and scanned at 254 nm in TLC III scanner.

#### Experimental animals husbandry

Male and female Sprague–Dawley rats, (130-160g) obtained from Central Animal Facility, Sri Ramachandra University, Chennai, India were used for the study. Animals were housed in individually in polypropylene cages in a ventilated room (air cycles: 15/min; 70:30 exchange ratio) under an ambient temperature of 22±2°C and 40–65% relative humidity, with a 12-h light/dark artificial photoperiod. They were provided with food (Nutrilab Rodent, Tetragon Chemie Pvt Ltd, India) and purified water *ad libitum*. All the animals were acclimatized at least for 7 days to the laboratory conditions prior to experimentation. Guidelines of “Guide for the Care and Use of Laboratory Animals” (Institute of Laboratory Animal Resources, National Academic Press 1996; NIH publication number #85-23, revised 1996) were strictly followed throughout the study. Institutional Animal Ethical Committee (IAEC), Sri Ramachandra University, Chennai, India approved the study (IAEC/XIX/SRU/133/2010).

#### Acute oral toxicity study

The acute oral toxicity test was performed following the guidelines of Organization for Economic Co-operation and Development (OECD) for testing of chemicals, TG 423 (adopted – December, 2001) with minor modifications [13]. Six female rats (nulliparous and non-pregnant; 140-160g body weight) were randomised into two groups (3 per group) viz., control and test groups. Control group received 0.5% carboxy methyl cellulose (prepared in double distilled water) as vehicle at a dose volume of 10ml/kg b. wt. whilst the test group received single oral dose of 2000 mg/kg b. wt. of NK (10ml/kg b. wt. in 0.5% CMC) via gastric intubation. All the experimental animals were observed for mortality and clinical signs of toxicity (general behaviour, respiratory pattern, cardiovascular signs, motor activities, reflexes and changes in skin and fur texture) at 30 min, 1, 2 and 4 hours and thereafter once a day for the next 14 days following vehicle or NK administration. Body weights were recorded once a week. On day 15, the overnight fasted animals (water allowed) were euthanized using CO_2_ euthanasia chamber and subjected to gross pathological examination of all the major internal organs such as brain, heart, lung, liver, kidney, spleen, adrenals and sex organs. LD_50_ cut-off value of NK was determined in accordance with Globally Harmonised System of Classification and Labelling of chemicals [14].

#### Repeated dose 28-day oral toxicity study

A 28-day repeated oral toxicity study was performed according to the OECD guideline, TG 407 (Revised - 18 December 2007) with minor modifications [15]. In Siddha practice, 3g/day of NK was recommended for the treatment of vitiligo in adult humans. The rat dose of 270mg/kg was arrived from the human dose based on body surface area conversion [16]. In the present study, NK was administered at three dose levels i.e., at 300, 600 and 900 mg/kg/day. Both sexes of Sprague Dawley rats (140-160g) were divided into 6 groups with 10 animals (5 males + 5 females) in each. Group I served as control and received 0.5% CMC as vehicle orally via gastric intubation at a dose volume of 10 ml/kg b. wt. Group II, III and IV received NK at 300, 600 and 900 mg/kg/day, p.o, respectively (10ml/kg b. wt. in 0.5% CMC), for a period of 28 days via gastric intubation. In order to determine the reversibility or recovery from toxic effects, if any, the satellite groups were preset. Group V served as satellite control (received vehicle) and group VI served as treatment satellite group which received NK at 900 mg/kg/day, p.o for a period of 28 days. The satellite groups were scheduled for follow-up observations for the next 14 days without vehicle or NK administration.

All the experimental animals were observed for mortality and morbidity twice a day, till the completion of treatment. Clinical observations were made once daily to detect signs of toxicity, preferably at the same time(s) in each day (1h after vehicle or NK administration). The focus of the observations was the same as described above for the acute toxicity study. Body weights of the animals were recorded once in a week. The amounts of food and water given and their remnants on the next day were measured to calculate the difference, which was regarded as daily consumption and the data were expressed as 7 days cumulative value.

At the end of the stipulated treatment period, the overnight fasted (water allowed) animals were anaesthetized, blood samples were collected by retro-orbital puncture in heparinised (for haematological and biochemical analysis) and non-heparinised tubes (for serum electrolytes). Haematological parameters such as haemoglobin (HGB), red blood cell count (RBC), white blood cell count (WBC), Hematocrit (HCT), platelet count (PLT), mean corpuscular volume (MCV), mean corpuscular hemoglobin (MCH), mean corpuscular haemoglobin concentration (MCHC), mean platelet volume (MPV), Plateletcrit (PCT) and red cell indices were measured using fully automated haematology analyzer (PE 6000). Plasma biochemical parameters such as glucose, total cholesterol (TC), triglycerides (TG), total protein (TP), albumin, serum glutamic oxaloacetic transaminase (SGOT), serum glutamic pyruvic transaminase (SGPT), lactate dehydrogenase (LDH), alkaline phosphatase (ALP), γ-glutamyl transpeptidase (γ-GT), bilirubin, creatinine (CRE) and blood urea nitrogen (BUN) were measured using diagnostic kits (Accurex, India) in a semi-automatic biochemical analyser (Star 21^plus^, India). Serum electrolytes such as sodium, potassium, chloride, total calcium and pH were estimated in a fully automated electrolyte analyzer (Cornley acculyte-5P, India).

#### Histopathology

Necropsy was done in all animals on day 29 except the satellite groups for which it was done on day 42. After blood collection, all the animals were euthanized for gross pathological examinations of all major internal organs. Organs such as brain, eyes, spinal cord, lymph nodes lung (right and left), prostate, thyroid gland, heart, stomach, liver, kidney, adrenals, thymus, spleen, intestine, muscle, bone, ovary, uterus, testis and urinary bladder were collected from all the animals for histopathology. The organs such as brain, heart, liver, spleen, kidneys, adrenals, thymus, testis/ovaries, epididymis were weighed and relative organ weights were calculated. However, it is planned to perform histopathological examination for the control and high dose group initially, if any histopatholgical findings were observed with high dose group, the low and mid dose groups were to be studied. The organs were fixed in 10% neutral buffered formalin, trimmed and a 5μ thickness of tissue sections were stained with hematoxylin and eosin for histopathological investigation.

#### Statistical analysis

Data were expressed in mean ± standard error mean (SEM). Mean difference between the control and treatment groups were analysed by Student ‘t’ test for acute toxicity and one way ANOVA followed by Tukey’s multiple comparison as posthoc test for 28-day repeated toxicity using GraphPad prism 5.0. *p* value ≤ 0.05 was considered as significance.

## Results

### Standardisation of NK

**Figure 1:**
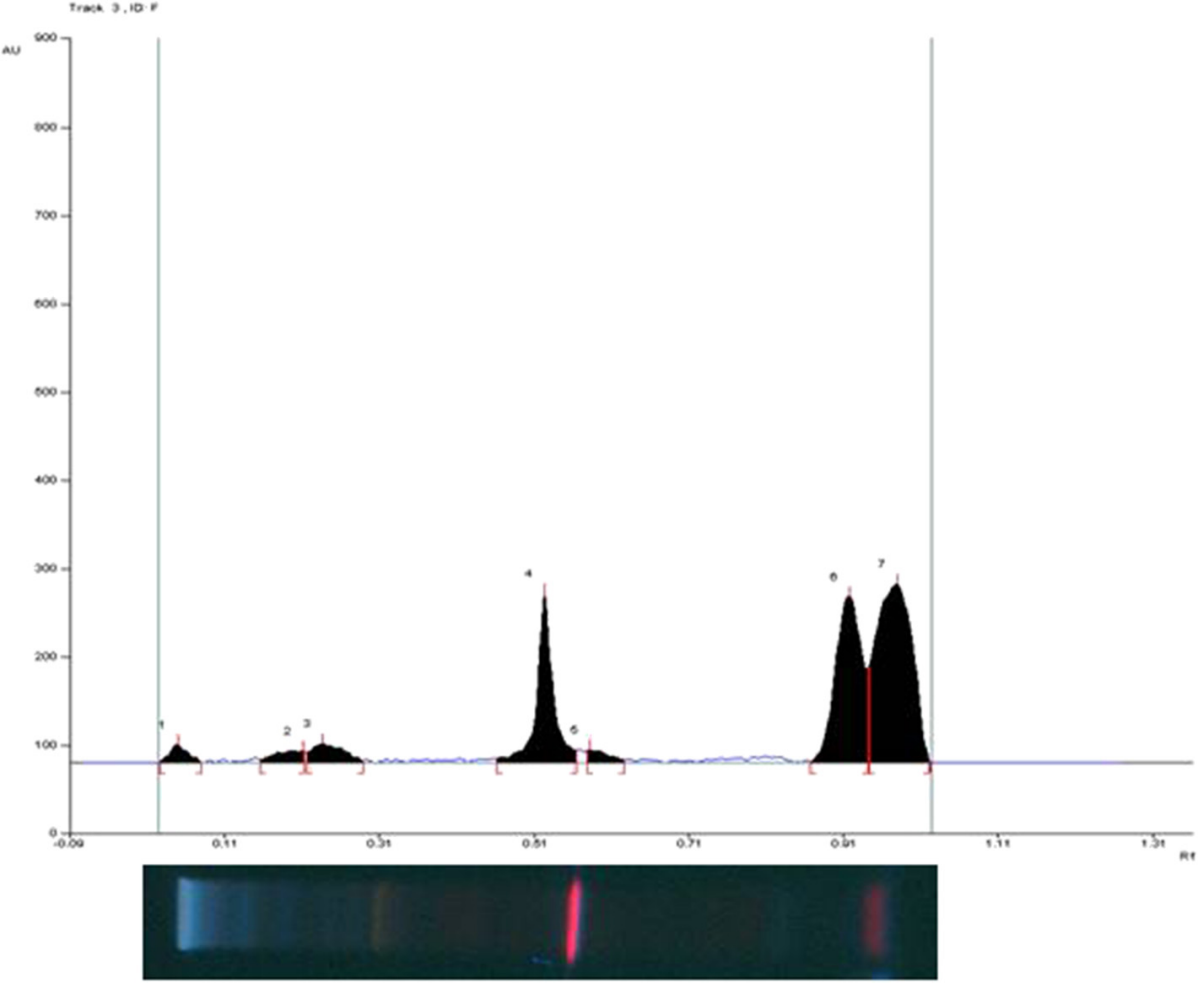
HPTLC Fingerprint of Nuna Kadugu.

Phytochemical qualitative screening of NK showed the presence different constituents like phenol, tannins, flavones and quinines (Table 1). Quantitative presence of total phenol, tannins and flavonoids contents of NK were found to be 0.56, 0.23 and 0.17% w/w, respectively (Table 2). Aflatoxins (B1, B2, G1 and G2) and heavy metals (lead, cadmium, mercury and arsenic) contents (Table 3) were found to be within the permissible limits recommended in safety standards for traditional medicines by Department of AYUSH, Ministry of Health and Family Welfare, Government of India [17]. HPTLC chromatogram of NK and photo documentation at 254 nm (Figure 1) revealed 7 peaks in the given chromatographic condition.

**Table 1:** Qualitative analysis of phytochemicals in Nuna kadugu

**S.No**	**Phytochemical**	**NK**
1.	Phenol	++
2.	Tannins	+
3.	Saponins	–
4.	Glycosides	–
5.	Sugar	–
6.	Alkaloids	+++
7.	Quinones	–
8.	Flavones	+++
9.	Anthraquinones	–
10.	Triterpenoids	–

Note: S.No – Serial number; NK- Nuna Kadugu; + −mild, ++ − moderate,+++ − high, – - absent.

**Table 2:** **Secondary metabolites in *Nuna kadugu***

**Secondary metabolites**	***Nuna kadugu***	
	**mg/g extract**	**%w/w**
**Tannins**	2.34 ± 0.86	0.23±0.08
**Total phenols**	5.60 ± 2.11	0.56±0.02
**Flavonoids**	1.74 ± 0.67	0.17±0.07

**Table 3:** Aflatoxin and heavy metal contents of Nuna Kadugu (NK)

**Parameters**	**NK**	**Maximum Permissible Limit (as per AYUSH)**
**Aflatoxin (ppm)**		
B1	ND	0.5
B2	ND	0.1
G1	ND	0.5
G2	ND	0.1
**Heavy metals**		
Lead	0.34	10 ppm
Cadmium	0.02	0.3 ppm
Mercury	0.04	1 ppm
Arsenic	ND	3 ppm

ND: Not detected.

### Acute oral toxicity study

**Figure 2:**
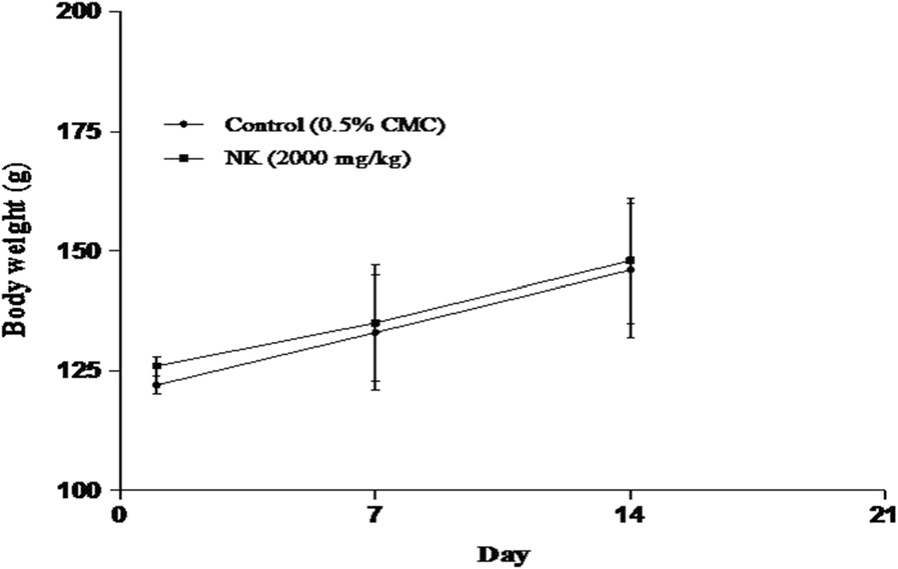
Effect of Nuna kadugu on body weight in control and NK rats - Acute oral toxicity study. Note: Values expressed as mean ± SEM (n=3).

There were no treatment related death or signs of toxicity developed in both the control and NK treated animals throughout the study. No significant difference in body weight gain was also observed (Figure 2). Further, there were no gross pathological abnormalities in both the groups. Thus the LD_50_ value was found to be greater than 2000mg/kg b.wt. With reference to the Globally Harmonised System of Classification and Labelling of chemicals, Nuna Kadugu can be classified as Category −5 and this provides direct relevance for protecting human and animal health.

### Repeated oral toxicity study

There were no treatment-related toxicity signs and mortality observed in both sexes of rats treated at 300, 600 and 900 mg/kg orally for a period of 28 days and in the satellite group of rats. No significant difference in body weight gain was observed between control and treated groups during the study (Table 4). Feed and water consumption of NK treated groups were found to be insignificant in both the sexes when compared to the 0.5% CMC treated rats (Table 5 and 6). Hematological parameters such as haemoglobin, red blood cells, white blood cells, platelet count, mean corpuscular volume, mean corpuscular haemoglobin, mean corpuscular haemoglobin concentration, mean platelet volume and red cell indices were found to be well within the clinical range of rats [18] in experimental groups (Table 7). There were no significant difference in plasma biochemical profile such as glucose, total cholesterol, triglycerides, total protein, albumin, alkaline phosphatise, γ-glutamyl transpeptidase, bilirubin, lactate dehydrogenase, creatinine, blood urea nitrogen (Table 8) observed between control and treated groups. The levels of liver marker enzymes like SGOT and SGPT were found to be well within the clinical range of rats [18] in NK treated groups (Table 8). Serum electrolytes such as sodium, potassium, chloride, total calcium and pH were found to be normal between the experimental groups (Table 9). There were no significant differences in organ and relative organ weight of brain, heart, liver, spleen, kidneys, adrenals and testis/ovaries recorded between the control and NK groups (Table 10 and 11). In our study, we performed hisopathological examinations in control and high dose group - brain, liver, lung (right and left), prostate, spleen, heart, spinal cord, lymph nodes, kidney, adrenals and other organs and they were revealed no abnormalities (Table 12). Hence we didn’t performed histopathology examination of low and mid dose groups. Representative histopathological photographs of brain, eyes, spinal cord, lung, lymph nodes thyroid gland, heart, stomach, liver, kidney, adrenals, thymus, spleen, intestine, muscle, bone, ovary, uterus, testis and urinary bladder of control, high dose and satellite groups (Fig not shown). There were no hematological, biochemical and histoptahological alterations observed with NK administration even at 900 mg/kg/day in rats for a period of 28 days compared to control. The No-Observed Adverse Effect Level (NOAEL) of NK was estimated to be greater than 900 mg/kg/day in rats. Hence, it can be concluded that Nuna Kadugu is safe for oral administration. .

**Table 4:** **Effect of *Nuna kadugu *on body weight gain in experimental rats - repeated oral toxicity study**

**Treatment**		**Sex**			** (g)**			
			**1**^**st**^**Week**	**2**^**st**^**Week**	**3**^**st**^**Week**	**4**^**st**^**Week**	**5**^**th**^**Week**	**6**^**th**^**Week**
**Control (0.5% CMC)**		M(n=5)	156.20±6.78	171.60±12.41	183.80±15.09	187.60±14.26		
		F (n=5)	155.4±10.3	157±7.75	166.6±4.80	169.6±4.54		
		MF(n=10)	155.8±5.82	164.3±7.32	175.2±8.00	178.6±7.67		
**NK**	**(300 mg/kg p.o/day)**	M(n=5)	159.2±6.64	178.6±10.67	189.4±14.7	193.6±13.92		
		F (n=5)	143.0±2.16	148.4±3.64	157.4±3.45	160.0±3.49		
		MF(n=10)	151.10±4.26	163.50±7.32	173.40±8.90	176.80±8.78		
	**(600 mg/kg p.o/day)**	M(n=5)	144.0±10.46	163.2±9.52	166.6±10.29	177.00±14.90		
		F (n=5)	142.40±10.27	159.40±11.85	163.00±10.13	177.80±11.06		
		MF(n=10)	143.20±6.89	161.30±7.19	164.80±6.83	177.40±8.74		
	**(900 mg/kg p.o/day)**	M(n=5)	149.00±8.91	168.60±10.34	190.00±12.12	195.20±12.24		
		F (n=5)	139.60±5.23	146.80±4.67	157.20±6.86	161.60±6.99		
		MF(n=10)	144.3±5.11	157.7±6.46	173.6±8.54	178.40±8.68		
**Satellite group**								
**Control (0.5% CMC)**		M(n=5)	141.00±12.54	164.80±16.47	169.80±17.46	174.60±17.57	178.40±20.52	185.00±18.43
		F (n=5)	139.80±3.63	147.80±4.91	147.80±4.30	148.60±4.17	155.80±2.70	160.40±2.78
		MF(n=10)	140.4±6.15	156.3±8.66	158.8±9.23	161.6±9.55	167.1±10.46	172.7±9.69
**NK (900 mg/kg p.o/day)**		M(n=5)	152.6±11.56	170.2±12.50	181.2±8.25	179.6±13.95	182.2±12.85	188.2±9.68
		F (n=5)	149.60±8.46	164.80±8.35	173.00±11.18	176.00±10.78	179.00±9.55	178.60±12.75
		MF(n=10)	151.1±6.77	167.5±7.14	177.1±6.69	177.8±8.33	180.6±7.56	183.4±7.77

Values expressed as mean ± SEM; g – gram; Significance with Tukey’s test following one way ANOVA is evaluated as ^*^p < 0.05 and ^**^p < 0.01 vs control group.

**Table 5:** **Effect of *Nuna kadugu *on feed intake in experimental rats - repeated oral toxicity study**

**Treatment**		**Sex**			** (g)**			
			**1**^**st**^**Week**	**2**^**nd**^**Week**	**3**^**st**^**Week**	**4**^**st**^**Week**	**5**^**th**^**Week**	**6**^**th**^**Week**
**Control (0.5% CMC)**		M(n=5)	80.60±3.61	82.20±7.44	89.40±7.86	101.00±1.64		
		F (n=5)	83.40±6.08	90.00±5.42	87.00±6.94	90.20±11.06		
		MF(n=10)	82.00±3.37	86.10±4.5	88.20±4.96	95.60±5.57		
**NK**	**(300 mg/kg p.o/day)**	M(n=5)	93.40±8.76	88.60±9.09	88.80±9.4	93.60±6.71		
		F (n=5)	76.20±2.71	81.60±4.41	87.40±4.01	97.00±7.84		
		MF(n=10)	84.80±5.19	85.10±4.90	88.10±4.86	95.30±4.90		
	**(600 mg/kg p.o/day)**	M(n=5)	87.40±5.64	93.00±4.60	92.80±4.12	96.60±5.27		
		F (n=5)	73.40±4.98	78.60±4.32	85.80±4.6	92.80±3.81		
		MF(n=10)	80.40±4.24	85.80±3.82	89.30±3.16	94.70±3.13		
	**(900 mg/kg p.o/day)**	M(n=5)	79.20±4.07	76.00±3.7	79.80±4.19	97.60±2.36		
		F (n=5)	85.80±11.83	92.80±8.76	102.00±5.30	90.60±11.38		
		MF(n=10)	82.50±6.00	84.40±5.30	90.90±4.88	94.10±5.60		
**Satellite group**								
**Control (0.5% CMC)**		M(n=5)	81.80±7.79	86.20±9.47	85.80±9.97	80.80±8.80	100.60±8.49	99.40±14.08
		F (n=5)	82.00±7.13	89.40±5.87	92.20±1.46	109.80±1.02	98.00±10.21	99.20±7.62
		MF(n=10)	81.90±4.98	87.80±5.28	89.00±4.87	95.306.39±	99.30±6.28	99.30±7.55
**NK (900 mg/kg p.o/day)**		M(n=5)	82.40±6.93	89.00±7.50	82.00±5.02	103.60±4.74	98.00±8.63	99.20±6.88
		F (n=5)	81.40±5.99	81.20±3.71	93.80±4.76	91.00±9.73	100.40±3.88	99.80±9.44
		MF(n=10)	81.90±4.32	85.10±4.15	87.90±5.02	97.30±5.52	99.20±4.48	99.50±5.51

Values expressed as mean ± SEM; g – gram; Significance with Tukey’s test following one way ANOVA is evaluated as ^*^p < 0.05 and ^**^p < 0.01 vs control group.

**Table 6:** **Effect of *Nuna kadugu *on water consumption in experimental rats - repeated oral toxicity study**

**Treatment**		**Sex**			** (ml)**			
			**1**^**st**^**Week**	**2**^**nd**^**Week**	**3**^**st**^**Week**	**4**^**st**^**Week**	**5**^**th**^**Week**	**6**^**th**^**Week**
**Control (0.5% CMC)**		M(n=5)	100.80±12.45	101.00±15.37	100.40±6.96	100.80±11.34		
		F (n=5)	98.40±5.53	101. 00±11.05	101.80±12.68	101.40±13.95		
		MF(n=10)	99.60±6.43	101.00±8.92	101.10±6.82	101.10±8.48		
**NK**	**(300 mg/kg p.o/day)**	M(n=5)	99.40±11.42	100. 20±9.61	101.20±16.83	100.60±13.67		
		F (n=5)	101.60±5.71	101.40±11.28	100.60±9.09	101.60±11.51		
		MF(n=10)	100.50±6.03	100.80±6.99	100.90±9.02	101.10±8.42		
	**(600 mg/kg p.o/day)**	M(n=5)	98.80±11.89	101.80±9.05	101.00±19.24	100.80±14.37		
		F (n=5)	100.00±7.29	100.80±8.00	102.20±17.30	100.40±11.21		
		MF(n=10)	99.40±6.58	101.30±5.70	101.60±12.20	100.60±8.59		
	**(900 mg/kg p.o/day)**	M(n=5)	99.80±9.61	100.40±5.35	100.20±9.44	98.80±7.00		
		F (n=5)	100.20±12.48	99.80±6.97	99.60±6.10	101.20±16.49		
		MF(n=10)	100.00±7.43	100. 10±4.14	99.90±5.30	100.00±8.45		
**Satellite group**								
**Control (0.5% CMC)**		M(n=5)	101.60±8.35	102.60±7.12	100.80±15.63	102.40±13.55	101.40±10.62	100.40±19.44
		F (n=5)	99.40±4.15	100.20±5.84	100.00±10.43	99.00±5.55	100.40±8.44	101.60±18.25
		MF(n=10)	100.50±4.41	101.40±4.36	100.40±8.86	100.70±6.93	100.90±6.40	101.00±12.57
**NK (900 mg/kg p.o/day)**		M(n=5)	101.60±8.39	101.40±10.63	101.60±14.66	101.20±4.04	102.40±11.78	101.60±10.80
		F (n=5)	99.40±13.01	99.80±8.26	100.60±11.29	101.60±11.71	101.00±14.32	102.20±12.29
		MF(n=10)	100.50±7.31	100.60±6.35	101.10±08.72	101.40±5.84	101.70±8.74	101.90±7.71

Values expressed as mean ± SEM; ml – milleliters; Significance with Tukey’s test following one way ANOVA is evaluated as ^*^p < 0.05 and ^**^p < 0.01 vs control group.

**Table 7:** **Effect of *Nuna kadugu *on hematological parameters in experimental rats - repeated oral toxicity study**

**Treatment**		**Sex**	**Hematological parameters**						
			**WBC (10**^**3**^**/uL)**	**RBC(10**^**6**^**/uL)**	**Hgb (%)**	**HCT (%)**	**MCV (fL)**	**MCH(pg)**	**MCHC(g/dl)**
**Control (0.5% CMC)**		M(n=5)	11.28±3.69	6.73±1.21	13.52±1.93	33.54±6.88	48.94±1.46	21.10±2.30	43.84±5.80
		F (n=5)	18.28±1.27	6.83±1.35	16.48±0.64	35.74±7.15	53.60±2.03	36.42±16.08	36.48±2.89
		MF(n=10)	15.97±2.01	6.39±0.89	14.85±1.11	33.01±4.81	50.81±1.53	29.30±8.00	39.95±4.46
**NK**	**(300 mg/kg p.o/day)**	M(n=5)	12.12±2.39	6.10±0.99	13.02±1.88	30.92±5.18	50.80±0.75	21.82±1.66	43.28±3.57
		F (n=5)	13.50±1.89	7.69±0.77	14.10±1.20	38.80±3.62	49.70±1.32	19.48±0.65	36.681.84±
		MF(n=10)	12.81±1.45	6.89±0.65	13.56±1.07	34.86±3.25	50.25±0.74	20.65±0.93	44.02±3.59
	**(600 mg/kg p.o/day)**	M(n=5)	12.68±2.39	6.54±0.29	13.20±0.82	33.40±1.24	48.54±4.69	19.76±0.72	44.82±4.15
		F (n=5)	14.08±1.35	8.03±0.58	15.64±2.01	40.08±3.40	45.54±3.01	19.52±1.20	46.50±5.15
		MF(n=10)	13.38±1.32	7.28±0.40	14.42 ±1.10	36.74±2.04	47.04±2.68	19.64±0.66	45.43±4.26
	**(900mg/kg p.o/day)**	M(n=5)	17.36±1.26	6.68±1.40	15.40±1.51	30.96±7.05	48.54 ±2.18	22.10±2.08	42.46±2.51
		F (n=5)	14.14±1.46	7.24±0.67	16.46±1.96	33.18±2.70	45.64±2.17	23.80±1.82	45.78±4.29
		MF(n=10)	15.75±1.05	6.96±0.74	15.93±1.18	32.07±3.58	47.09±1.53	22.95±1.33	44.12±2.41
**Satellite group**									
**Control (0.5% CMC)**		M(n=5)	18.18±2.30	8.67±0.55	18.48±0.90	42.82±1.73	47.08±1.97	19.48±0.57	40.94±2.13
		F (n=5)	12.18±2.11	5.19±0.96	14.22±1.95	26.48±5.90	50.02±1.54	29.72±5.70	60.34±10.29
		MF(n=10)	15.18±1.78	6.93±0.78	16.35±1.24	34.65±3.98	48.55±1.28	24.60±3.20	50.64±6.71
**NK (900 mg/kg p.o/day)**		M(n=5)	15.88±0.90	8.78±0.38	15.60±1.03	42.74±2.32	46.16±3.17	18.24±1.07	45.16±3.05
		F (n=5)	8.84±1.26	6.63±1.14	14.42±2.22	33.46±6.08	50.10±0.77	23.30±1.67	42.36±3.60
		MF(n=10)	12.36±1.38	7.70±0.67	15.01±1.17	38.10±3.44	48.13±1.67	20.77±1.26	43.76±2.27
**Treatment**			**Sex**	**Hematological parameters**					
				**RDW-CV (%)**	**PLT (10**^**3**^**/uL)**	**MPV (fL)**	**PDW (%)**	**PCT (%)**	**P-LCR (%)**
**Control (0.5% CMC)**			M(n=5)	14.32±0.60	324.80±57.03	10.00±0.37	9.30±0.24	0.18±0.02	20.64±2.01
			F (n=5)	14.16±0.24	386.20±16.74	10.84±0.19	8.98±0.22	0.17±0.01	26.48±1.15
			MF(n=10)	14.15± 0.33	372.90± 36.83	10.68 ± 0.19	8.65 ± 0.25	0.17 ± 0.02	23.87 ± 1.22
**NK**	**(300 mg/kg p.o/day)**		M(n=5)	13.70±0.54	351.00±45.03	10.34±0.78	9.02±0.60	0.11±0.01	20.08±2.83
			F (n=5)	13.54±0.70	393.40±74.87	10.62±0.39	9.34±0.52	0.11±0.01	22.80±1.24
			MF(n=10)	13.62± 0.42	372.20± 41.79	10.48 ± 0.41	9.18± 0.38	0.11± 0.02	21.44± 1.53
	**(600 mg/kg p.o/day)**		M(n=5)	16.58±2.62	369.40±65.75	8.96±0.74	8.80±0.28	0.15±0.02	22.53±1.11
			F (n=5)	14.04±0.38	377.40±66.43	10.52±0.21	8.88±0.24	0.15±0.02	20.66±1.33
			MF(n=10)	15.31 ± 1.32	373.40± 44.08	9.71± 0.49	8.85± 0.19	0.15± 0.01	21.65± 1.62
	**(900mg/kg p.o/day)**		M(n=5)	13.46±1.10	378.00±4.32	8.56±1.39	9.56±0.39	0.13±0.03	19.98±1.30
			F (n=5)	14.90±0.57	366.00±60.78	10.640.87±	8.30±0.63	0.16±0.02	20.22±1.57
			MF(n=10)	14.18± 0.63	372.00± 28.79	9.60± 0.85	8.93±0.41	0.15± 0.02	20.10± 0.96
**Satellite group**									
**Control (0.5% CMC)**			M(n=5)	14.08±0.56	377.20±46.72	10.76±0.19	8.32±0.40	0.15±0.02	22.54±1.31
			F (n=5)	13.86±0.89	369.20±29.87	10.44±0.42	8.70±0.22	0.20±0.08	25.18±1.51
			MF(n=10)	13.97± 0.50	373.20±26.17	10.60±0.22	8.51±0.22	0.17±0.04	23.86±1.04
**NK (900 mg/kg p.o/day)**			M(n=5)	15.70±1.34	373.60±39.33	10.56±0.76	7.98±0.51	0.11±0.01	28.20±0.86
			F (n=5)	15.00±0.58	371.20±34.98	10.09±0.59	9.70±0.36	0.10±0.01	25.70±1.76
			MF(n=10)	15.35± 0.70	372.40±24.82	10.32±0.46	8.84±0.41	0.11±0.01	26.95±1.01

Values expressed as mean ± SEM; n=10 (5/sex); Significance with Tukey’s test following one way ANOVA is evaluated as ^*^p < 0.05 and ^**^p < 0.01 vs control group. Haemoglobin (HGB), red blood cell count (RBC), white blood cell count (WBC), Hematocrit (HCT), mean corpuscular volume (MCV), mean corpuscular hemoglobin (MCH), mean corpuscular haemoglobin concentration (MCHC).

Values expressed as mean ± SEM; Significance with Tukey’s test following one way ANOVA is indicated as ^*^p < 0.05 and ^**^p < 0.01 vs control group. Mean platelet volume (MPV), Plateletcrit (PCT) and red cell indices, platelet (PLT), RDW – red blood cell distribution Width, PDW – Platelet distribution Width.

**Table 8:** **Effect of *Nuna kadugu *on biochemical parameters of experimental Sprague Dawley rats in repeated oral toxicity study**

**Treatment**		**Sex**	**Biochemical parameters**						
			**Glucose (mg/dl)**	**Cholesterol (mg/dl)**	**Triglycerides (mg/dl)**	**T. Protein (gm%)**	**Albumin (gm%)**	**SGOT (IU/l)**	**SGPT (IU/l)**
**Control (0.5% CMC)**		M(n=5)	128.90 ±12.51	88.01 ±5.69	45.18 ±8.56	4.63 ±0.28	2.27 ±0.13	30.40 ±4.04	33.92 ±3.38
		F (n=5)	134.24 ±5.13	86.64 ±7.32	50.50 ±9.00	4.70 ±0.34	2.38 ±0.23	29.40 ±4.12	43.16 ±7.41
		MF(n=10)	131.57±6.44	87.32±4.38	47.84±5.92	4.67±0.21	2.33±0.13	29.90±2.73	38.54±4.14
**NK**	**(300 mg/kg p.o/day)**	M(n=5)	131.16 ±9.57	87.98 ±10.81	51.20 ±11.66	4.90 ±0.13	2.43 ±0.15	29.92 ±5.31	43.83 ±6.02
		F (n=5)	130.31 ±11.64	87.74 ±13.2	44.79 ±3.18	5.01 ±0.15	2.34 ±0.23	29.90 ±3.93	34.19 ±4.42
		MF(n=10)	130.74±7.11	87.86±8.05	47.99±5.80	4.96±0.09	2.39±0.13	29.91±3.11	39.01±3.87
	**(600 mg/kg p.o/day)**	M(n=5)	128.35 ±12.39	89.26 ±3.32	50.57 ±1.53	4.82 ±0.27	2.22 ±0.29	29.10 ±3.69	40.99 ±4.52
		F (n=5)	132.61 ±15.43	85.92 ±10.74	44.74 ±3.50	4.78 ±0.49	2.20 ±0.23	31.22 ±4.86	38.66 ±3.67
		MF(n=10)	130.48±9.35	87.59±5.33	47.66±2.05	4.80±0.26	2.21±0.18	30.16±2.90	39.83±2.77
	**(900mg/kg p.o/day)**	M(n=5)	130.18 ±2.79	85.73 ±14.31	47.91 ±6.91	4.95 ±0.50	2.33 ±0.30	30.20 ±4.21	38.56 ±6.79
		F (n=5)	130.58 ±13.84	88.77 ±14.81	47.05 ±5.99	4.66 ±0.28	2.24 ±0.13	31.08 ±6.96	41.00 ±8.08
		MF(n=10)	130.38±6.66	87.25±9.72	47.48±4.31	4.80±0.28	2.28±0.15	30.64±3.84	39.78±4.99
**Satellite group**									
**Control (0.5% CMC)**		M(n=5)	134.32 ±8.85	89.59 ±10.96	47.55 ±3.73	4.92 ±0.32	2.34 ±0.25	31.14 ±1.17	39.68 ±4.37
		F (n=5)	127.92 ±12.83	85.61 ±10.53	48.65 ±2.67	5.05 ±0.45	2.12 ±0.22	29.54 ±4.74	39.01 ±2.98
		MF(n=10)	131.12±7.42	87.60±7.19	48.10±2.17	4.99±0.26	2.23±0.16	30.34±2.32	39.35±2.50
**NK (900 mg/kg p.o/day)**		M(n=5)	131.55 ±4.12	87.57 ±5.61	46.79 ±5.22	4.80 ±0.31	2.20 ±0.13	24.96 ±4.73	39.88 ±4.38
		F (n=5)	132.35 ±14.32	86.61 ±13.93	49.42 ±4.24	4.71 ±0.33	2.42 ±0.19	35.36 ±3.85	38.66 ±7.83
		MF(n=10)	131.95±7.02	87.09±7.08	48.11±3.20	4.75±0.21	2.31±0.12	30.16±3.36	39.27±4.24
**Treatment**			**Sex**	**Biochemical parameters**					
				**LDH (IU/L)**	**ALP (IU/L)**	**γ-GT (IU/L)**	**Bilirubin (mg/dl)**	**Creatinine (mg%)**	**BUN (mg/dl)**
**Control (0.5% CMC)**			M(n=5)	178.20±9.72	329.00±46.89	2.39±0.23	0.83±0.03	0.43±0.05	19.07±0.63
			F (n=5)	180.60±12.33	286.80±51.41	2.11±0.14	0.90±0.03	0.31±0.03	20.18±1.43
			MF(n=10)	179.40±7.41	307.90±33.55	2.25±0.13	0.87±0.02	0.37±0.03	19.63±0.76
**NK**	**(300 mg/kg .o/day)**		M(n=5)	179.60±16.64	307.22±18.45	2.12±0.19	0.54±0.27	0.28±0.02	19.06±3.10
			F (n=5)	178.20±12.08	310.06±14.29	2.10±0.20	1.00±0.08	0.35±0.03	18.08±1.28
			MF(n=10)	178.90±9.69	308.64±11.01	2.11±0.13	0.77±0.15	0.31±0.02	18.57±1.59
	**(600 mg/kg p.o/day)**		M(n=5)	181.40±7.28	306.74±16.29	2.03±0.23	0.84±0.05	0.34±0.04	18.56±2.11
			F (n=5)	179.80±11.51	311.32±32.42	1.96±0.20	0.84±0.06	0.34±0.04	20.26±1.33
			MF(n=10)	180.60±6.42	309.03±25.89	1.99±0.14	0.84±0.04	0.34±0.03	19.41±1.21
	**(900mg/kg p.o/day)**		M(n=5)	178.20±14.03	310.82±35.07	2.04±0.21	0.99±0.04	0.37±0.03	19.62±1.65
			F (n=5)	183.60±8.59	306.64±31.52	2.13±0.22	0.97±0.02	0.30±0.03	18.55±2.00
			MF(n=10)	180.90±7.81	308.73±35.56	2.09±0.14	0.98±0.02	0.33±0.02	19.09±1.24
**Satellite group**									
**Control (0.5% CMC)**			M(n=5)	180.60±10.13	288.66±20.91	2.01±0.25	0.92±0.06	0.32±0.03	19.88±0.86
			F (n=5)	181.60±16.71	330.98±50.39	2.06±0.28	0.93±0.05	0.34±0.03	20.78±1.10
			MF(n=10)	181.10±9.21	309.82±26.66	2.04±0.18	0.93±0.04	0.33±0.02	20.33±0.68
**NK (900 mg/kg p.o/day)**			M(n=5)	180.20±13.43	310.80±33.66	2.28±0.29	0.96±0.13	0.38±0.04	21.21±2.33
			F (n=5)	181.20±14.59	308.42±36.41	1.85±0.23	0.930.04±	0.31±0.03	20.48±1.60
			MF(n=10)	180.70±9.35	309.61±23.38	2.06±0.19	0.94±0.06	0.35±0.03	20.85±1.34

Values expressed as mean ± SEM; Significance with Tukey’s test following one way ANOVA is evaluated as ^*^p < 0.05 and ^**^p < 0.01 vs control group. SGOT – Serum Glutamate oxaloacetate transaminase; SGPT – Serum Glutamate pyruvate transaminase.

Values expressed as mean ± SEM; n=10 (5/sex); Significance with Tukey’s test following one way ANOVA is evaluated as ^*^p < 0.05 and ^**^p < 0.01 vs control group. LDH – Lactate dehydrogenase; ALP- Alkaline Phosphatase; GGT- Gamma Glutamyl Transferase; BUN – Blood Urea Nitrogen.

**Table 9:** **Effect of *Nuna kadugu *on serum electrolytes of experimental Sprague Dawley rats in repeated oral toxicity study**

**Treatment**		**Sex**	**Serum electrolytes (mmol/l)**				
			**Sodium**	**Potassium**	**Chloride**	**Total Calcium**	**pH**
**Control (0.5% CMC)**		M(n=5)	136.46±0.59	4.68±0.19	99.48±1.32	2.38±0.02	7.35±0.09
		F (n=5)	140.09±0.86	4.06±0.10	100.94±1.95	2.45±0.02	7.42±0.13
		MF(n=10)	138.28±0.78	4.37±0.14	100.21±1.14	2.42±0.02	7.39±0.08
**NK**	**(300 mg/kg p.o/day)**	M(n=5)	139.03±0.73	4.40±0.15	99.49±1.14	2.33±0.08	7.41±0.09
		F (n=5)	137.76±0.31	4.02±0.08	99.20±0.49	2.47±0.02	7.40±0.06
		MF(n=10)	138.39±0.43	4.21±0.10	99.34±0.59	2.40±0.05	7.40±0.05
	**(600 mg/kg p.o/day)**	M(n=5)	138.12±0.70	4.35±0.12	99.53±0.33	2.47±0.01	7.38±0.11
		F (n=5)	138.03±1.04	4.08±0.12	100.27±0.53	2.38±0.03	7.39±0.05
		MF(n=10)	138.07±0.59	4.21±0.09	99.90±0.32	2.42±0.02	7.39±0.06
	**(900mg/kg p.o/day)**	M(n=5)	142.58±1.20	4.23±0.13	102.82±1.09	2.41±0.02	7.40±0.09
		F (n=5)	134.75±2.47	4.19±0.12	98.15±1.03	2.41±0.05	7.36±0.10
		MF(n=10)	138.67±1.84	4.21±0.08	100.49±1.05	2.41±0.03	7.38±0.06
**Satellite group**							
**Control (0.5% CMC)**		M(n=5)	147.40±1.68	4.25±0.04	100.43±1.62	2.36±0.07	7.39±0.09
		F (n=5)	132.23±6.90	4.19±0.05	101.02±1.62	2.44±0.07	7.39±0.04
		MF(n=10)	139.82±4.19	4.22±0.03	100.73±1.09	2.40±0.05	7.39±0.04
**NK (900 mg/kg p.o/day)**		M(n=5)	140.23±1.15	4.25±0.07	102.02±1.83	2.35±0.09	7.40±0.13
		F (n=5)	139.57±0.98	4.20±0.08	99.79±3.32	2.42±0.07	7.40±0.15
		MF(n=10)	139.90 ± 0.72	4.23± 0.05	100.90 ± 1.82	2.39± 0.05	7.40 ± 0.09

Values expressed as mean ± SEM; n=10 (5/sex); Significance with Tukey’s test following one way ANOVA is evaluated as ^*^p < 0.05 and ^**^p < 0.01 vs control group.

**Table 10:** **Effect of *Nuna kadugu *on organ weight of experimental Sprague Dawley rats in repeated oral toxicity study**

**Treatment**		**Sex**	**Organ Weight (gms)**				
			**Kidneys**	**Adrenals**	**Thymus**	**Testis/Ovaries**	**Epididymis**
**Control (0.5% CMC)**		M(n=5)	1.65±0.13	0.05±0.01	0.48±0.01	2.06±0.42	1.43±0.03
		F (n=5)	1.34±0.13	0.04±0.00	0.48±0.03	0.08±0.02	-
		MF(n=10)	1.49±0.10	0.04± 0.00	0.48±0.01	-	-
**NK**	**(300 mg/kg p.o/day)**	M(n=5)	1.79±0.12	0.05±0.01	0.48±0.01	2.09±0.17	1.41±0.03
		F (n=5)	1.35±0.04	0.05±0.01	0.49±0.02	0.32±0.02	-
		MF(n=10)	1.57±0.09	0.05±0.00	0.48±0.01	-	-
	**(600 mg/kg p.o/day)**	M(n=5)	1.61±0.10	0.04±0.00	0.47±0.02	1.96±0.19	1.47±0.03
		F (n=5)	1.36±0.07	0.04±0.00	0.48±0.01	0.08±0.01	-
		MF(n=10)	1.49±0.07	0.04±0.00	0.47±0.01	-	-
	**(900mg/kg p.o/day)**	M(n=5)	1.64±0.14	0.04±0.00	0.47±0.01	2.22±0.38	1.49±0.03
		F (n=5)	1.37±0.08	0.05±0.01	0.52±0.03	0.09±0.01	-
		MF(n=10)	1.51±0.09	0.05±0.00	0.49±0.02	-	-
**Satellite group**							
**Control (0.5% CMC)**		M(n=5)	1.51±0.12	0.05±0.01	0.46±0.02	1.98±0.43	1.41±0.03
		F (n=5)	1.34±0.07	0.05±0.00	0.49±0.02	0.10±0.01	-
		MF(n=10)	1.42±0.07	0.05±0.00	0.48±0.01	-	-
**NK (900 mg/kg p.o/day)**		M(n=5)	1.68±0.10	0.05±0.00	0.490.02	2.04±0.31	1.54±0.02
		F (n=5)	1.35±0.05	0.05±0.00	0.51±0.03	0.12±0.00	
		MF(n=10)	1.51±0.08	0.05±0.00	0.50±0.02	1.98±0.43	

Values expressed as mean ± SEM; n=10 (5/sex); Significance with Tukey’s test following one way ANOVA is evaluated as ^*^p < 0.05 and ^**^p < 0.01 vs control group.

**Table 11:** **Effect of *Nuna kadugu *on relative organ weight of experimental Sprague Dawley rats in repeated oral toxicity study**

**Treatment**		**Sex**	**Relative organ weight (g %)**							
			**Brain**	**Heart**	**Liver**	**Spleen**	**Kidney**	**Adrenal**	**Sex organs**	
									**Testis**	**Ovaries**
**Control (0.5% CMC)**		M (n=5)	0.99±0.06	0.38±0.02	3.53±0.22	0.47±0.03	0.88±0.03	0.03±0.00	1.07±0.15	-
		F (n=5)	1.05±0.04	0.42±0.43	4.21±0.35	0.49±0.02	0.79±0.08	0.02±0.00	-	0.05±0.01
		MF (n=10)	1.02±0.03	0.40±0.02	3.87±0.23	0.48±0.02	0.84±0.04	0.02±0.00	-	-
**NK**	**(300 mg/kg p.o/day)**	M (n=5)	0.97±0.08	0.41±0.01	3.70±0.14	0.54±0.11	0.93±0.06	0.02±0.00	1.12±0.15	-
		F (n=5)	1.10±0.05	0.40±0.02	3.79±0.28	0.42±0.04	0.85±0.03	0.03±0.00	-	0.07±0.01
		MF (n=10)	1.04±0.05	0.40±0.01	3.74±0.15	0.48±0.06	0.89±0.03	0.03±0.00	-	-
	**(600 mg/kg p.o/day)**	M (n=5)	1.06±0.12	0.40±0.03	3.99±0.75	0.43±0.06	0.96±0.15	0.02±0.00	1.16±0.20	-
		F (n=5)	1.00±0.04	0.39±0.03	3.45±0.12	0.38±0.03	0.77±0.03	0.02±0.00	-	0.05±0.01
		MF (n=10)	1.03±0.06	0.40±0.02	3.72±0.37	0.40±0.03	0.86±0.08	0.02±0.00	-	-
	**(900mg/kg p.o/day)**	M (n=5)	0.97±0.06	0.38±0.02	3.63±0.41	0.48±0.04	0.84±0.03	0.02±0.00	1.11±0.16	-
		F (n=5)	1.14±0.04	0.40±0.01	3.90±0.19	0.52±0.06	0.85±0.02	0.03±0.00	-	0.06±0.01
		MF (n=10)	1.05±0.05	0.39±0.01	3.76±0.22	0.50±0.03	0.84±0.02	0.03±0.00	-	-
										**Satellite group**										
**Control (0.5% CMC)**		M (n=5)	1.03±0.10	0.41±0.02	3.93±0.26	0.41±0.03	0.83±0.05	0.03±0.00	1.07±0.19	-	
		F (n=5)	1.08±0.04	0.36±0.01	3.91±0.25	0.46±0.05	0.84±0.04	0.03±0.00	-	0.07±0.01	
		MF (n=10)	1.05±0.05	0.39±0.01	3.92±0.17	0.44±0.03	0.83±0.03	0.03±0.00	-	-	
**NK (900 mg/kg p.o/day)**		M (n=5)	0.99±0.04	0.41±0.04	4.06±0.27	0.49±0.03	0.89±0.04	0.03±0.00	1.07±0.12	-	
		F (n=5)	1.02±0.03	0.39±0.04	3.41±0.44	0.39±0.04	0.77±0.07	0.03±0.00	-	0.07±0.01	
		MF (n=10)	1.02±0.02	0.40±0.03	3.74±0.27	0.44±0.03	0.83±0.04	0.03±0.00	-	-	

Values expressed as mean ± SEM; n=10 (5/sex); Significance with Tukey’s test following one way ANOVA is evaluated as ^*^p < 0.05 and ^**^p < 0.01 vs control group.

**Table 12:** Histopathological investigation of control and NK treated animals for 28 days

**Tissue**	**Treatment**	**Male**	**Female**
**Brain, Eyes**	Control (0.5% CMC)	Normal	Normal
	NK (900 mg/kg p.o/day)	Normal	Normal
	Satellite - NK (900 mg/kg p.o/day)	Normal	Normal
**Lung (right and left), Prostate, Thyroid gland, Thymus**	Control (0.5% CMC)	Normal	Normal
	NK (900 mg/kg p.o/day)	Normal	Normal
	Satellite - NK (900 mg/kg p.o/day)	Normal	Normal
**Spinal Cord, Lymph Nodes, Muscle, Bone**	Control (0.5% CMC)	Normal	Normal
	NK (900 mg/kg p.o/day)	Normal	Normal
	Satellite - NK (900 mg/kg p.o/day)	Normal	Normal
**Heart, Liver**	Control (0.5% CMC)	Normal	Normal
	NK (900 mg/kg p.o/day)	Normal	Normal
	Satellite - NK (900 mg/kg p.o/day)	Normal	Normal
**Stomach, Intestine**	Control (0.5% CMC)	Normal	Normal
	NK (900 mg/kg p.o/day)	Normal	Normal
	Satellite - NK (900 mg/kg p.o/day)	Normal	Normal
**Kidney, Adrenals, Urinary bladder**	Control (0.5% CMC)	Normal	Normal
	NK (900 mg/kg p.o/day)	Normal	Normal
	Satellite - NK (900 mg/kg p.o/day)	Normal	Normal
**Sex organ (Testis/Epididymis)**	Control (0.5% CMC)	Normal	-
	NK (900 mg/kg p.o/day)	Normal	-
	Satellite - NK (900 mg/kg p.o/day)	Normal	-
**Sex organ (Uterus /ovaries/)**	Control (0.5% CMC)	-	Normal
	NK (900 mg/kg p.o/day)	-	Normal
	Satellite - NK (900 mg/kg p.o/day)	-	Normal

## Discussion

Herbal medicines have attained greater importance as an alternative to conventional therapy. To optimize the safe use of a plant-based medicine, one should take into account their historical applications on humans and animals as well as toxicity evaluation of the medicinal herbs and their active components [19]. Many screening methods are employed to determine the safety and efficacy of these herbal medicines and also to establish the active component of the herbal products [20]. Siddha system of medicine is an ancient medical system of Dravidian origin which is prevalent mainly in Southern parts of India, especially in Tamil Nadu. Nuna (*Morinda pubescens*), is a medicinal herb used in the treatment of vitiligo by many Siddha practitioners in Tamil Nadu. However, the scientific validation of its safety and efficacy has not been established so far. The present study gives detailed information on the toxicological profile of Nuna Kadugu by acute and repeated oral toxicity studies in rats.

Since NK is in clinical use for vitiligo treatment for more than 10 years, a limit test was performed in acute oral toxicity study. According to the OECD test guideline 423 when there is information in support of low or non-toxicity and immortality nature of the test material, then the limit test at the highest starting dose level (2000 mg/kg body weight) was conducted. There were no mortality and toxicity signs observed at 2000mg/kg. Nuna Kadugu can be classified under category-5 and LD_50_ value was greater than 2000mg/kg in accordance with Globally Harmonised System of Classification and Labelling of chemicals and this provides us a direct relevance for protecting human and animal health. Therefore, it can be concluded that Nuna Kadugu when administered at single dose is non-toxic and can be used safely in oral formulations.

A 28-day repeated oral toxicity study was performed followed OECD test guideline 407 (Revised 18 December 2007) in both male and female Sprague Dawley rats. Since examination of clinical signs plays major role in toxicological testing [21], mortality and morbidity were recorded twice a day throughout the study. NK did not produce any alterations in feed and water consumption and this reveals that it did not adversely affect the basic metabolic processes of the experimental animals. The haemopoietic system serves as important target for toxic chemicals and is a sensitive index for pathological conditions both in humans and animals [22]. In the present study, treatment with NK did not produce any alteration in haematological parameters (i.e. RBC, WBC, haemoglobin, haematocrit etc.), which indicate that NK did not affect blood cells nor their production. Clinical biochemistry and hematological data holds significant role in determining the toxicity induced by drugs [18]. Transaminases (SGOT and SGPT) are good indicators of liver function and biomarkers to predict the possible toxicity of drugs [23]. Any elevation pertaining to these enzymes indicate their outflow into the blood stream due to damage in liver parenchymal cells.

There were no changes in the SGPT and SGOT levels which reveal that NK did not affect liver function/or metabolism. In the present study, there were no treatment related abnormalities in renal function and other biochemical parameters suggesting that NK is non-toxic. Similarly, the serum electrolyte levels were found to be well within the clinical range of rats which reflects that NK has no adverse effect on ionic homeostasis. Histopathological studies provide supportive evidence for biochemical and haematological observations [24]. The relative organ weights were found to be non-significant between the control and NK treated rats. No abnormality was recorded with respect to gross or histopathological examinations of all organs examined. Since there were no signs of toxicity with respect to hematology, clinical chemistry, organ weight, gross and histopathological examinations noted in NK satellite group, it can be inferred that NK will not produce delayed onset of toxicity. Based on these results, the No Observed Adverse Effect Level (NOAEL) of “Nuna Kadugu” is greater than 900 mg/kg/day.

## Conclusion

In accordance with Globally Harmonised System of Classification and Labelling of chemicals, NK can be classified as Category 5. Based on 28 day repeated dose toxicity study, NOAEL of NK is greater than 900 mg/kg/day. The present investigation substantiates, at least in part, the safety of NK, which was found to be in line with the long history of its use in Siddha system of medicine.

## Competing interest

Authors declare that they have no competing interests.

## Authors’ contributions

RSR designed and drafted the manuscript; NP, RS, HS, KTM performed the experiment and assisted in manuscript preparation; RJP performed pathology; JRV animals health monitoring, clinical signs assessment and manuscript preparation; CSB designed the study; KM conceived the study, ST coordinated the study. All authors read and approved the final manuscript.

## Pre-publication history

The pre-publication history for this paper can be accessed here:

http://www.biomedcentral.com/1472-6882/12/190/prepub
